# Presepsin: gelsolin ratio, as a promising marker of sepsis-related organ dysfunction: a prospective observational study

**DOI:** 10.3389/fmed.2023.1126982

**Published:** 2023-05-05

**Authors:** Dániel Ragán, Péter Kustán, Zoltán Horváth-Szalai, Balázs Szirmay, Attila Miseta, Gábor Woth, Tamás Kőszegi, Diána Mühl

**Affiliations:** ^1^Department of Laboratory Medicine, University of Pécs Medical School, Pécs, Hungary; ^2^Department of Anesthesiology and Intensive Therapy, University of Pécs Medical School, Pécs, Hungary; ^3^János Szentágothai Research Center, University of Pécs, Pécs, Hungary; ^4^Department of Anesthesia, Intensive Care and Pain Medicine, Klinik Ottakring, Vienna, Austria; ^5^National Laboratory on Human Reproduction, University of Pécs, Pécs, Hungary

**Keywords:** sepsis-3, organ dysfunction, prognosis, presepsin, gelsolin, presepsin:gelsolin ratio, novel biomarker

## Abstract

**Introduction:**

We aimed to facilitate the diagnosis and prognosis of sepsis-related organ dysfunction through analyzing presepsin (PSEP) and gelsolin (GSN) levels along with a novel marker, the presepsin:gelsolin (PSEP:GSN) ratio.

**Methods:**

Blood samples were collected from septic patients at the intensive care unit (ICU) at three time points (T1-3): T1: within 12 h after admission; T2: second day morning; T3: third day morning. Sampling points for non-septic ICU patients were T1 and T3. PSEP was measured by a chemiluminescence-based POCT method while GSN was determined by an automated immune turbidimetric assay. Data were compared with routine lab and clinical parameters. Patients were categorized by the Sepsis-3 definitions. PSEP:GSN ratio was evaluated in major sepsis-related organ dysfunctions including hemodynamic instability, respiratory insufficiency and acute kidney injury (AKI).

**Results:**

In our single center prospective observational study, 126 patients were enrolled (23 control, 38 non-septic and 65 septic patients). In contrast to controls, significantly elevated (*p* < 0.001) admission PSEP:GSN ratios were found in non-septic and septic patients. Regarding 10-day mortality prediction, PSEP:GSN ratios were lower (*p* < 0.05) in survivors than in non-survivors during follow-up, while the prognostic performance of PSEP:GSN ratio was similar to widely used clinical scores (APACHE II, SAPS II, SOFA). PSEP:GSN ratios were also higher (*p* < 0.001) in patients with sepsis-related AKI than septic non-AKI patients during follow-up, especially in sepsis-related AKI patients needing renal replacement therapy. Furthermore, increasing PSEP:GSN ratios were in good agreement (*p* < 0.001) with the dosage and the duration of vasopressor requirement in septic patients. Moreover, PSEP:GSN ratios were markedly greater (*p* < 0.001) in patients with septic shock than in septic patients without shock. Compared to septic patients requiring oxygen supplementation, substantially elevated (*p* < 0.001) PSEP:GSN ratios were observed in septic patients with demand for mechanical ventilation, while higher PSEP:GSN ratios (*p* < 0.001) were also associated with extended periods of mechanical ventilation requirement in septic patients.

**Conclusion:**

PSEP:GSN ratio could be a useful complementary marker besides the routinely used SOFA score regarding the diagnosis and short term mortality prediction of sepsis. Furthermore, the significant increase of this biomarker may also indicate the need for prolonged vasopressor or mechanical ventilation requirement of septic patients. PSEP:GSN ratio could yield valuable information regarding the extent of inflammation and the simultaneous depletion of the patient’s scavenger capacity during sepsis.

**Clinical trail registration:**

NIH U.S. National Library of Medicine, ClinicalTrails.gov. Trial identifier: NCT05060679, (https://clinicaltrials.gov/ct2/show/NCT05060679) 23.03.2022, Retrospectively registered.

## Introduction

Sepsis is still a leading cause of mortality at the intensive care unit (ICU) despite the availability of modern treatment modalities (1, 2). Recent epidemiological studies suggest an increasing incidence along with a slightly decreasing mortality rate (3–5). As stated in the latest sepsis-3 definitions, sepsis is a life-threatening organ dysfunction caused by a dysregulated host response to infection (6).

Any vital organ system could be affected during the development of sepsis, therefore the most important manifestations of organ dysfunctions include hemodynamic instability, respiratory insufficiency, acute kidney injury (AKI), acute liver failure, thrombocytopenia and altered mental state (6, 7). The currently used Acute Physiology and Chronic Health Evaluation II (APACHE II), Simplified Acute Physiology Score II (SAPS II) and Sequential Organ Failure Assessment (SOFA) scores have major advantages regarding the prognosis of critically ill patients. However, these clinical prediction scores may have limitations due to the heterogeneity of sepsis itself (6–9).

Serum procalcitonin (PCT) and high-sensitivity C-reactive protein (hs-CRP) are commonly utilized inflammatory markers during the clinical evaluation of septic patients, yet the role of biomarkers remains unspecified in the sepsis-3 definitions (6, 7). Apart from hs-CRP and PCT, approximately 200 promising sepsis biomarkers have been examined to date, however, no single marker had adequate sensitivity and specificity for the diagnosis and prognosis of sepsis (9–11). On the other hand, a multi-marker approach involving novel sepsis biomarkers (e.g., presepsin, IL-6) could be useful regarding this issue.

Presepsin (PSEP) is a 13-kDa soluble fragment of the 55-kDa cluster of differentiation marker protein 14 (CD14), which is the receptor for lipopolysaccharide (LPS) and LPS-binding protein complexes (12, 13). PSEP measurement was found to be valuable in the early diagnosis of sepsis and the evaluation of sepsis severity compared with other inflammatory conditions (e.g., trauma, burning, surgeries) (14, 15). According to several multicentric studies, the diagnostic cut-off levels of PSEP varied among 400–600 pg/ml for sepsis, while PSEP was also useful regarding the prognosis of septic patients (16–19). Furthermore, numerous studies showed increased PSEP concentrations in different conditions involving renal dysfunction (e.g., chronic kidney disease, sepsis-related AKI) (20–24).

Gelsolin (GSN; MW = 83 kDa) is an essential component of the so-called extracellular actin scavenger system, due to its protective role by sequestering of liberated actin in the circulation while also modulating the immune response (25–28). As a result, significantly lower serum GSN levels were detected in various inflammatory diseases (29–31). A previous study conducted in our institute also suggested that increased serum actin:GSN ratios correlated with higher mortality rates in patients with severe sepsis (32).

We hypothesized that the simultaneous measurement of PSEP and GSN levels could yield valuable information regarding the diagnosis and prognosis of sepsis and sepsis-related organ dysfunctions. Therefore, we investigated a new potential marker: the presepsin:gelsolin (PSEP:GSN) ratio.

The primary objectives of our study were the followings:

- Comparing PSEP:GSN ratios of control, non-septic and septic patients
- Analyzing the diagnostic performance of PSEP:GSN ratio in non-septic vs. septic patients
- Examining the 10-day mortality prediction of PSEP:GSN ratio in sepsis.

The secondary objectives of our study were as follows:

- Investigating PSEP:GSN ratio in sepsis-related hemodynamic instability based on the dosage and the duration of vasopressor requirement
- Analyzing PSEP:GSN ratio in sepsis-related respiratory insufficiency based on the requirement for oxygen supplementation vs. mechanical ventilation
- Evaluating PSEP:GSN ratio in the diagnosis of sepsis-related AKI.

## Materials and methods

### Study design

A previous study was performed in our institute investigating urinary actin in control, septic and sepsis-related AKI patients (33). Besides healthy control individuals, non-septic patients needing ICU hospitalization after major surgical interventions (e.g., esophageal or pancreatic cancer surgery, cardiac surgery) and acutely diagnosed septic patients were enrolled consecutively in our single center prospective observational study conducted between January 2018 and February 2020 at the Department of Anesthesiology and Intensive Therapy (Medical School, University of Pécs, Hungary). Detailed information was given to all patients or their next-of-kin regarding our study protocol while written consent was obtained from all. Exclusion criteria were patients under 18 years of age, unobtainable or withdrawn consent, end-stage renal disease requiring chronic dialysis or kidney transplantation and patients with malignancies in palliative care. The study protocol was registered retrospectively at ClinicalTrials.gov (NCT05060679) and was approved by the Regional Research Ethics Committee of the University of Pécs (no. 4327.316-2,900/KK15/2011) conforming to the 7th revision of the Helsinki Declarations (2013).

Control individuals were recruited as outpatients from the Department of Ophthalmology (Medical School, University of Pécs, Hungary). Exclusion criteria were lack of consent, infectious disease, kidney disease or acute inflammation (hs-CRP >5 mg/L).

#### Definitions

- Sepsis: The diagnosis of sepsis was determined after admission based on the sepsis-3 definitions (6). Inclusion criteria for sepsis were the followings: a suspected or microbiologically confirmed infection and at least 1 vital organ dysfunction shown in increased SOFA score (>2). Non-septic patients also could have had elevated admission SOFA scores, yet these patients’ clinical status was not associated with the presence of an infection. Therapeutic approaches of sepsis were based on the international guidelines of the actual Surviving Sepsis Campaign (SSC) (7, 34).
- Sepsis-related hemodynamic instability: Following the classification of the SOFA score for the cardiovascular system, patients were categorized based on their worst daily values (monitored hourly) of vasopressor (mostly norepinephrine) requirement into low (≤0.1 μg/kg/min) and high (>0.1 μg/kg/min) dose groups, while patients were also divided based on shorter (≤5 consecutive days) and longer (>5 consecutive days) vasopressor requirement during ICU stay. Patients with septic shock were identified as stated in the sepsis-3 definitions (6).
- Sepsis-related respiratory insufficiency: Patients were categorized based on their requirement for oxygen supplementation (e.g., face mask (FiO_2_ ≥ 50%), high-flow nasal oxygen therapy) and mechanical ventilation (invasive ventilation after endotracheal intubation), while the latter group was also divided based on shorter (≤7 consecutive days) and longer (>7 consecutive days) requirement for mechanical ventilation during ICU stay (6, 7). Patients needing mechanical ventilation were further divided based on the development of (at least) moderate acute respiratory distress syndrome (ARDS) according to the Berlin definition (35).
- Sepsis-related AKI: Patients with elevated serum creatinine levels and/or decreased urine output within 24 h after admission were considered to have AKI based on the Kidney Disease Improving Global Outcomes (KDIGO) classification (36). Therapeutic interventions of sepsis-related AKI followed the aforementioned SSC and KDIGO guidelines (7, 34, 36).
- Multiple organ dysfunction syndrome (MODS): Septic patients were regarded to have MODS if they developed at least 2 or more vital organ dysfunctions (e.g., hemodynamic instability, respiratory insufficiency, altered mental state, AKI, acute liver failure, thrombocytopenia) during follow-up based on significantly elevated SOFA scores (at least ≥2 points for every organ dysfunction).

Patients with sepsis-related acute liver failure and thrombocytopenia were not investigated (although initially planned), as these complications occurred only in <20% of the septic study population.

As the majority of mechanically ventilated septic patients received propofol or dexmedetomidine sedation during the early stages of respiratory failure, we had limitations regarding the accurate assessment of the patients’ level of consciousness using the Glasgow Coma Scale.

First-day values of SAPS II, APACHE II and SOFA scores were calculated for assessing disease severity. Patients were categorized as survivors and non-survivors using 10-day mortality data.

### Sampling

Similarly to our previous study, blood samples were collected from septic patients at the ICU at three time points (T1-3): T1: within 12 h after admission; T2: second day morning of follow-up; T3: third day morning of follow-up (33). Sampling points for non-septic patients were the first (T1) and third (T3) postoperative morning. Arterial blood was obtained from every non-septic and septic patient from arterial catheter using plastic blood collection tubes with accelerator gel (5 ml) for serum samples, glucose/lactate and EDTA-anticoagulated tubes (4 ml) for plasma samples (BD Vacutainer, Franklin Lakes, NJ, United States). Not more than one sample (venous blood) was collected from controls. Anticoagulated blood samples were centrifuged immediately (10 min, 1,500 g) while for native blood, tubes were centrifuged after coagulation (10 min, 1,500 g), then plasma and serum aliquots were stored without preservatives at −70°C until analysis.

### Laboratory analysis

Serum parameters including total protein (se-TP), albumin, bilirubin, kidney function markers (se-urea, se-creatinine) along with plasma lactate, platelet count (PLT), and inflammatory parameters (white blood cell count (WBC), hs-CRP, PCT) were measured using automated routine procedures at our accredited laboratory (Department of Laboratory Medicine, Medical School, University of Pécs, Hungary; NAH-9-0008/2021). Serum gelsolin (GSN) was measured by an automated immune turbidimetric assay [Cobas 8,000/c502 module (Roche Diagnostics GmbH, Mannheim, Germany)] developed and validated in our laboratory (37, 38).

### Determination of plasma presepsin levels and presepsin:gelsolin ratio

PSEP concentrations were measured using an automated Point of Care instrument (PATHFAST; LSI Medience Corporation, Tokyo, Japan) based on a chemiluminescent enzyme immunoassay technique with a detection range of 20–20,000 pg/ml (39). Tests were performed according to the manufacturer’s instructions and the performance of the method was checked by bi-level controls. PSEP:GSN ratio was calculated as the ratio of PSEP to GSN concentrations.

### Statistical analysis

The IBM SPSS Statistics for Windows, Version 22 (IBM Corp., NY, United States) software was used for statistical analysis. Since our data did not show normal distribution by the Kolmogorov–Smirnov and Shapiro–Wilk tests, we performed non-parametric tests. The control, non-septic and septic patient groups were compared using Chi-square or Fischer’s exact test for qualitative data and Mann–Whitney U or Kruskal–Wallis tests for quantitative data. Friedman’s ANOVA with *post hoc* Dunn tests along with Wilcoxon tests were carried out to compare the quantitative data of different time points in every patient group. Diagnostic and prognostic performance of laboratory and clinical parameters were evaluated by receiver operating characteristic (ROC) curves. Spearman’s rank correlation test was applied for investigating relationships between quantitative variables. Quantitative data were presented as medians and interquartile ranges (IQR) while qualitative data as frequencies and percentages (%). Values of *p* < 0.05 were considered as statistically significant. The significance level was adjusted according to the Bonferroni correction during the analysis of multiple comparisons. The MedCalc Statistical Software, Version 20 (MedCalc Software Ltd., Ostend, Belgium) was used for performing the DeLong tests when comparing the individual ROC curves with each other.

## Results

### Patients’ demographic and laboratory data

In the present study, a total of 126 patients (23 control, 38 non-septic, 65 septic) were enrolled consecutively. In addition, 37 more patients (11 control, 7 non-septic, 19 septic) were excluded during the recruitment period of our study. Admission demographic, laboratory and clinical data are shown in Table 1. A moderate difference (*p* < 0.017) was found between the patient groups regarding age and some of the listed of comorbidities. A significant difference (*p* < 0.001) was observed between the control, non-septic and septic patient groups in se-TP, se-albumin, hs-CRP, PSEP and GSN levels along with PSEP:GSN ratios. Admission values of se-urea, se-creatinine, WBC and PLT were also different (*p* < 0.017) as well in the non-septic and septic groups compared with those of the controls. APACHE II, SAPS II and SOFA scores along with PCT levels were higher (*p* < 0.001) in septic patients than in non-septic patients. Major therapeutic requirements of 38 non-septic patients were the followings: all patients received adequate fluid resuscitation, yet 23 (60.5%) had temporary low dose vasopressor requirement; 36 (94.7%) needed oxygen supplementation, 2 (5.3%) received temporary mechanical ventilation, 5 (13.2%) had temporary AKI-1 stage kidney injury and nobody required renal replacement therapy (RRT) during follow-up.

**Table 1:** Patients’ admission demographic, laboratory and clinical data.

	Control (*n* = 23)	Non-sepsis (*n* = 38)	Sepsis (*n* = 65)	*p* value
Age (years)	52 (48–56)	64 (56–72)	68 (57–73)	<0.001^a,b^
Males, *n* (%)	13 (56.5)	26 (68.4)	43 (66.2)	0.409
Major comorbidities, *n* (%)				
Cardiovascular disease	10 (43.5)	33 (86.8)	51 (78.5)	0.002^a,b^
Type-2 diabetes mellitus	5 (21.7)	12 (24.9)	19 (29.2)	0.488
Chronic kidney disease	0 (0)	3 (7.9)	8 (12.3)	0.284
Pulmonary disease	2 (8.7)	11 (28.9)	12 (18.5)	0.218
Immunological disease	1 (4.3)	2 (5.2)	2 (3.1)	0.625
Malignancy	0 (0)	10 (26.3)	18 (27.7)	0.009^a,b^
Admission laboratory data				
se-TP (g/L)	76.1 (72.2–77.7)	51.7 (47.3–57.4)	47.6 (40.3–50.3)	<0.001^a,b,c^
se-albumin (g/L)	49.2 (46.9–51.1)	34.3 (29.2–38.5)	23.4 (19.5–27.7)	<0.001^a,b,c^
se-urea (mmol/L)	4.6 (4.0–5.5)	4.4 (3.5–5.8)	15.1 (9.9–24.9)	<0.001^b,c^
se-creatinine (μmol/L)	76 (70–86)	73 (62–99)	159 (99–285)	<0.001^b,c^
se-bilirubin (μmol/L)	11.2 (6.7–15.9)	7.3 (5.4–14.1)	9.7 (5.1–21.9)	0.169
WBC (G/L)	7.2 (6.4–7.9)	14.1 (12.1–16.2)	16.4 (10.6–22.7)	<0.001^a,b^
PLT (G/L)	262 (249–300)	165 (132–205)	197 (139–301)	0.004^a,b^
hs-CRP (mg/L)	1.3 (0.6–2.5)	102.8 (72.6–141.6)	284.2 (172.8–382.1)	<0.001^a,b,c^
PCT (ng/ml)	–	0.7 (0.3–2.1)	10.9 (4.5–48.7)	<0.001^c^
PSEP (pg/ml)	127 (89.5–159)	329 (209.5–442.5)	1,185 (501–3,073)	<0.001^a,b,c^
GSN (mg/L)	78.5 (75.1–89.1)	34.3 (28.7–40.3)	11.2 (6.1–20.8)	<0.001^a,b,c^
PSEP:GSN ratio (ng/mg)	1.7 (1.1–2.1)	9.9 (5.5–14.3)	105.9 (41.1–322.7)	<0.001^a,b,c^
Admission clinical data				
APACHE II score	–	7 (6–8)	20 (15–24)	<0.001^c^
SAPS II score	–	20 (17–26)	46 (36–55)	<0.001^c^
SOFA score	–	5.5 (3–7)	10 (8–12)	<0.001^c^
ICU treatment days	–	2 (1–3)	8 (4–14)	<0.001^c^

Continuous variables are shown as median (25th–75th percentiles) and categorical variables are expressed as a number (percentage). Mann–Whitney U and Chi-square tests were used for data comparison between patient groups. Level of significance was adjusted to *p* < 0.017 (according to the Bonferroni correction). Superscript lowercase letters refer to *post-hoc* analyses: ^a^*p* < 0.017 between control and non-sepsis; ^b^*p* < 0.017 between control and sepsis; ^c^*p* < 0.017 between non-sepsis and sepsis groups. TP: total protein; WBC: white blood cell count; PLT: platelet count; hs-CRP: high-sensitivity C-reactive protein; PCT: procalcitonin; PSEP: presepsin; GSN: gelsolin; PSEP:GSN: presepsin:gelsolin ratio; APACHE II: Acute Physiology and Chronic Health Evaluation II score; SAPS II: Simplified Acute Physiology Score II; SOFA: Sequential Organ Failure Assessment score; ICU: intensive care unit.

### Septic patients’ clinical data

Major therapeutic requirements of 65 septic patients were the followings: 54 (83.1%) needed vasopressor support, 48 (73.8%) were treated with mechanical ventilation [median (IQR) Horowitz quotient: 157 (117–207) mmHg], 17 (26.2%) received oxygen supplementation [median (IQR) Horowitz quotient: 333 (273–412) mmHg], 53 (81.5%) required hydrocortisone supplementation. Mechanically ventilated patients received propofol or dexmedetomidine sedation during the early stage of severe respiratory failure. Furthermore, only 11 (16.9%) septic patients developed liver failure, while also 11 (16.9%) septic patients had thrombocytopenia.

All of the 45 sepsis-related AKI patients were given adequate fluid resuscitation, however, 16 (35.5%) of them with the most severe condition also received – besides fluid replacement and vasopressor therapy – invasive hemodynamic monitoring (PiCCO). In addition, 7 (58.3%) AKI-1, 7 (53.8%) AKI-2 and 14 (70.0%) AKI-3 stage septic patients were treated with diuretics (mostly furosemide). Furthermore, 15 (75.0%) AKI-3 stage septic patients required some form of RRT: 6 (40.0%) received intermittent hemodialysis (IHD) and 9 (60.0%) were treated with continuous renal replacement therapy (CRRT).

Septic patients were further divided based on the occurrence of MODS. Relevant data of septic patients with MODS (*n* = 41) and without MODS (*n* = 24) are presented in Supplementary Data Sheet 1 and in Supplementary Table 1 as well.

### Monitoring presepsin:gelsolin ratio in control, non-septic and septic patients

**Figure 1:**
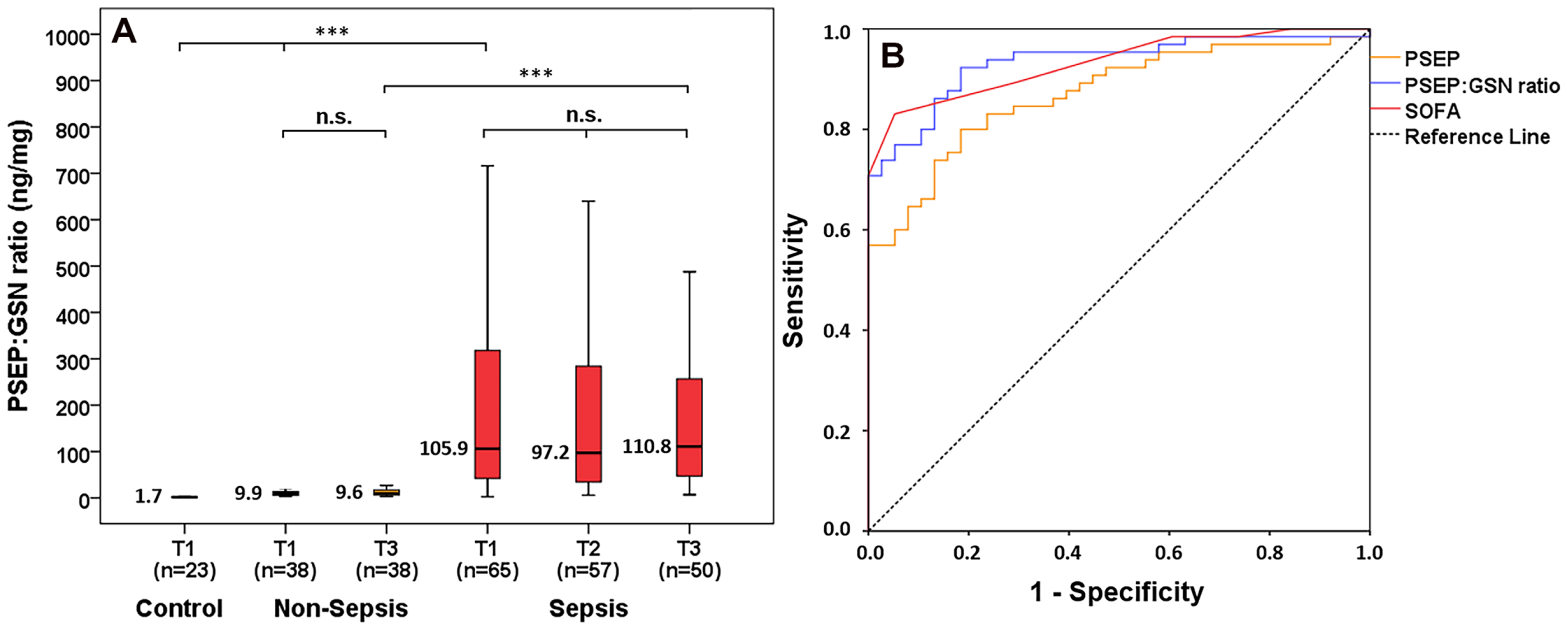
PSEP:GSN ratio in control, non-septic and septic patients. PSEP:GSN ratios of control, non-septic and septic patients during follow-up **(A)**. Receiver operating characteristic (ROC) curves of admission laboratory parameters for distinguishing non-sepsis from sepsis **(B)**. Time points: T1: within 12 h after admission; T2: second day; T3: third day. PSEP, presepsin; PSEP:GSN, presepsin:gelsolin ratio; PCT, procalcitonin; SOFA, Sequential Organ Failure Assessment score. n: sample number; n.s.: not significant. ***p < 0.001.

An elevating trend was found in PSEP:GSN ratios between the control and non-septic patients at T1 (median: 1.7 vs. 9.9 ng/mg, *p* < 0.001), while septic patients showed higher PSEP:GSN ratios than non-septic patients at T1 (median: 9.9 vs. 105.9 ng/mg, *p* < 0.001) and T3 (median: 9.6 vs. 110.8 ng/mg, *p* < 0.001). There was no significant change in the kinetics of PSEP:GSN ratios during follow-up regarding the non-septic (T1, T3 median: 9.9 vs. 9.6 ng/mg, *p* = 0.151) and septic (T1, T2, T3 median: 105.9 vs. 97.2 vs. 110.8 ng/mg, *p* = 0.487) patient groups (Figure 1A). The diagnostic performance of first-day PSEP:GSN ratios in sepsis was assessed using ROC analysis. For distinguishing all non-septic ICU patients from septic patients, area under the curve (AUC) value of first-day PSEP:GSN ratio (*p* < 0.001) was found to be acceptable in contrast to SOFA (*p* < 0.001) and PSEP (*p* < 0.001; Figure 1B; Table 2).

**Table 2:** Receiver operating characteristic (ROC) curve analysis of ICU patients.

Variable	AUC (95% CI)	Standard error*	Sens. (%)	Spec. (%)	Cut-off value	*p* value
Non-sepsis (*n* = 38) versus sepsis (*n* = 65)						
PSEP (pg/ml)	0.870 (0.803–0.937)	0.034	80.0	81.6	479.5	<0.001
PSEP:GSN ratio (ng/mg)	0.933 (0.886–0.981)	0.024	92.3	81.6	16.3	<0.001
SOFA score	0.933 (0.887–0.978)	0.023	83.1	94.7	7.5	<0.001
Comparison of ROC curves (DeLong test significance levels)						
PSEP versus PSEP:GSN (*p* = 0.007); PSEP versus SOFA (*p* = 0.049); PSEP:GSN versus SOFA (*p* = 0.988)						
Survivors (*n* = 47) versus non-survivors (*n* = 18) in sepsis (10-day mortality)						
PSEP (pg/ml)	0.683 (0.545–0.821)	0.071	72.2	59.6	1186.0	0.023
PSEP:GSN ratio (ng/mg)	0.719 (0.576–0.862)	0.073	72.2	70.2	161.2	0.007
APACHE II score	0.784 (0.659–0.908)	0.063	77.8	78.7	21.5	<0.001
SAPS II score	0.778 (0.660–0.897)	0.061	72.2	76.6	49.5	0.001
SOFA score	0.745 (0.619–0.827)	0.064	77.8	70.2	10.5	0.002
Comparison of ROC curves (DeLong test significance levels)						
PSEP versus PSEP:GSN (*p* = 0.351); PSEP versus APACHE II (*p* = 0.139); PSEP versus SAPS II (*p* = 0.197); PSEP versus SOFA (*p* = 0.403); PSEP:GSN versus APACHE II (*p* = 0.415); PSEP:GSN versus SAPS II (*p* = 0.483); PSEP:GSN versus SOFA (*p* = 0.763); APACHE II versus SAPS II (*p* = 0.915); APACHE II versus SOFA (*p* = 0.542); SAPS II versus SOFA (*p* = 0.556)						
Septic non-AKI (*n* = 20) versus sepsis-related AKI (*n* = 45)						
PSEP (pg/ml)	0.897 (0.820–0.974)	0.039	80.0	80.0	705.0	<0.001
PSEP:GSN ratio (ng/mg)	0.782 (0.670–0.894)	0.057	84.4	65.0	53.6	<0.001
se-creatinine (μmol/L)	0.925 (0.858–0.992)	0.034	88.9	90.0	139.5	<0.001
Comparison of ROC curves (DeLong test significance levels)						
PSEP versus PSEP:GSN (*p* = 0.015); PSEP versus se-creat (*p* = 0.524); PSEP:GSN versus se-creat (*p* = 0.018)						

Receiver operating characteristic (ROC) curve analysis of admission laboratory and clinical parameters for distinguishing non-sepsis from sepsis and predicting 10-day mortality in sepsis along with differentiating septic non-AKI from sepsis-related AKI. AUC, area under the curve; 95% CI, 95% confidence interval; Sens., sensitivity; Spec., specificity; ICU, intensive care unit; AKI, acute kidney injury; PSEP, presepsin; PSEP:GSN, presepsin:gelsolin ratio; PCT, procalcitonin; APACHE II, Acute Physiology and Chronic Health Evaluation II score; SAPS II, Simplified Acute Physiology Score II; SOFA, Sequential Organ Failure Assessment score. *DeLong et al., 1988.

### Usefulness of presepsin:gelsolin ratio regarding the 10-day mortality prediction in sepsis

**Figure 2:**
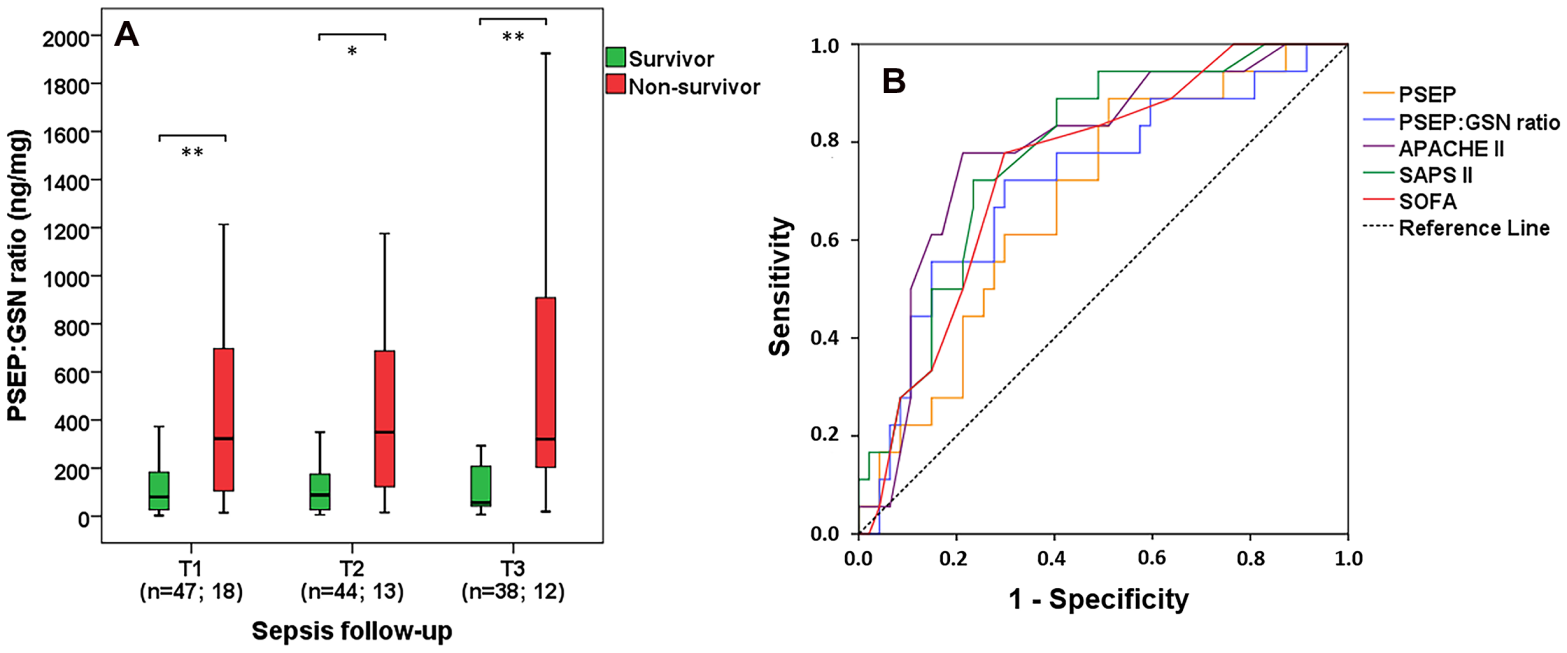
Survival data and predictive power of PSEP:GSN ratio. PSEP:GSN ratio in survivor and in non-survivor septic patients based on 10-day mortality during follow-up **(A)**. Receiver operating characteristic (ROC) curves of admission parameters for predicting 10-day mortality in sepsis **(B)**. Time points: T1: within 12 h after admission; T2: second day; T3: third day. PSEP, presepsin; PSEP:GSN, presepsin:gelsolin ratio; APACHE II, Acute Physiology and Chronic Health Evaluation II score; SAPS II, Simplified Acute Physiology Score II; SOFA, Sequential Organ Failure Assessment score. n: sample number. *p < 0.05; **p < 0.01.

PSEP:GSN ratios were significantly lower in survivors compared with non-survivors at T1 (median: 80.6 vs. 322.7 ng/mg, *p* = 0.007), T2 (median: 88.4 vs. 349.4 ng/mg, *p* = 0.01) and T3 (median: 56.3 vs. 320.6 ng/mg, *p* = 0.007) as well (Figure 2A). Regarding 10-day mortality prediction, AUC values of first-day PSEP:GSN ratio (*p* = 0.007) and PSEP (*p* = 0.023) were comparable to APACHE II (*p* < 0.001), SAPS II (*p* = 0.001) and SOFA (*p* = 0.002) scores (Figure 2B; Table 2).

### Presepsin:gelsolin ratio in sepsis based on requirements of vasopressor support

**Figure 3:**
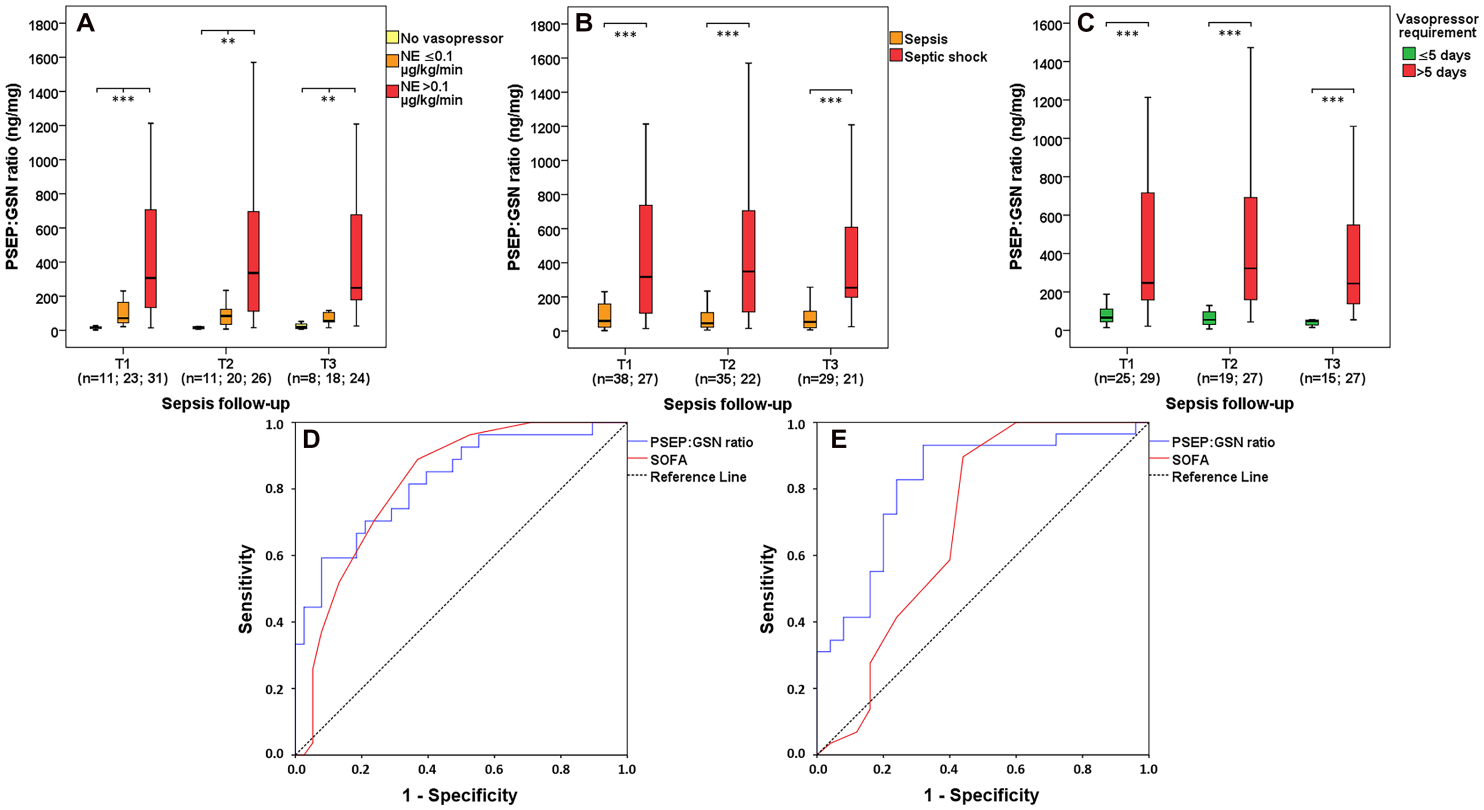
PSEP:GSN ratio in septic patients based on vasopressor requirement. PSEP:GSN ratios of septic patients with different doses of vasopressor requirement during follow-up **(A)**. PSEP:GSN ratios of patients with sepsis and septic shock **(B)** during follow-up. PSEP:GSN ratios of septic patients needing shorter (≤5 days) and longer (>5 days) vasopressor support during follow-up **(C)**. Receiver operating characteristic (ROC) curves of admission parameters for distinguishing sepsis from septic shock **(D)** along with discerning septic patients’ shorter (≤5 days) and longer (>5 days) vasopressor requirement **(E)**. Time points: T1: within 12 h after admission; T2: second day; T3: third day. NE, norepinephrine; PSEP, presepsin; PSEP:GSN, presepsin:gelsolin ratio; PCT, procalcitonin; SOFA, Sequential Organ Failure Assessment score. n: sample number. **p < 0.01; ***p < 0.001.

In contrast to septic patients with no vasopressor requirement, proportionately elevated PSEP:GSN ratios were found in septic patients with lower (≤0.1 μg/kg/min) and higher (>0.1 μg/kg/min) doses of norepinephrine requirement at T1 (median: 17.4 vs. 70.9 vs. 307.1 ng/mg, *p* < 0.001), T2 (median: 16.4 vs. 83.9 vs. 336.1 ng/mg, *p* = 0.001) and T3 (median: 19.1 vs. 54.5 vs. 249.1 ng/mg, *p* = 0.016; Figure 3A). Thus, patients with septic shock showed significantly increased PSEP:GSN ratios compared to septic patients without septic shock at T1 (median: 59.2 vs. 317.8 ng/mg, *p* < 0.001), T2 (median: 45.9 vs. 349.3 ng/mg, *p* < 0.001) and T3 (median: 53.2 vs. 254.1 ng/mg, *p* < 0.001; Figure 3B). Furthermore, septic patients with demand for vasopressor support longer than 5 consecutive days had substantially higher PSEP:GSN ratios than septic patients with shorter (≤5 days) vasopressor requirement at T1 (median: 66.7 vs. 247.4 ng/mg, *p* < 0.001), T2 (median: 54.9 vs. 323.1 ng/mg, *p* < 0.001) and T3 (median: 48.9 vs. 243.9 ng/mg, *p* < 0.001) as well (Figure 3C). For distinguishing all patients with septic shock from patients without septic shock, first-day AUC values were the following: PSEP:GSN ratio: 0.824 (*p* < 0.001); SOFA: 0.818 (*p* < 0.001). Derived cut-off values were: PSEP:GSN ratio: 161.2 ng/mg (sensitivity: 70.4%; specificity: 78.9%); SOFA: 10.5 (sensitivity: 70.4%; specificity: 76.3%; Figure 3D). For discerning septic patients with shorter (≤5 days) and longer (>5 days) vasopressor support, first-day AUC values were as follows: PSEP:GSN ratio: 0.821 (*p* < 0.001); SOFA: 0.698 (*p* = 0.013). Derived cut-off values were: PSEP:GSN ratio: 91.7 ng/mg (sensitivity: 93.1%; specificity: 68.0%); SOFA: 9.5 (sensitivity: 89.7%; specificity: 56.0%; Figure 3E).

### Presepsin:gelsolin ratio in sepsis based on requirements of respiratory support

**Figure 4:**
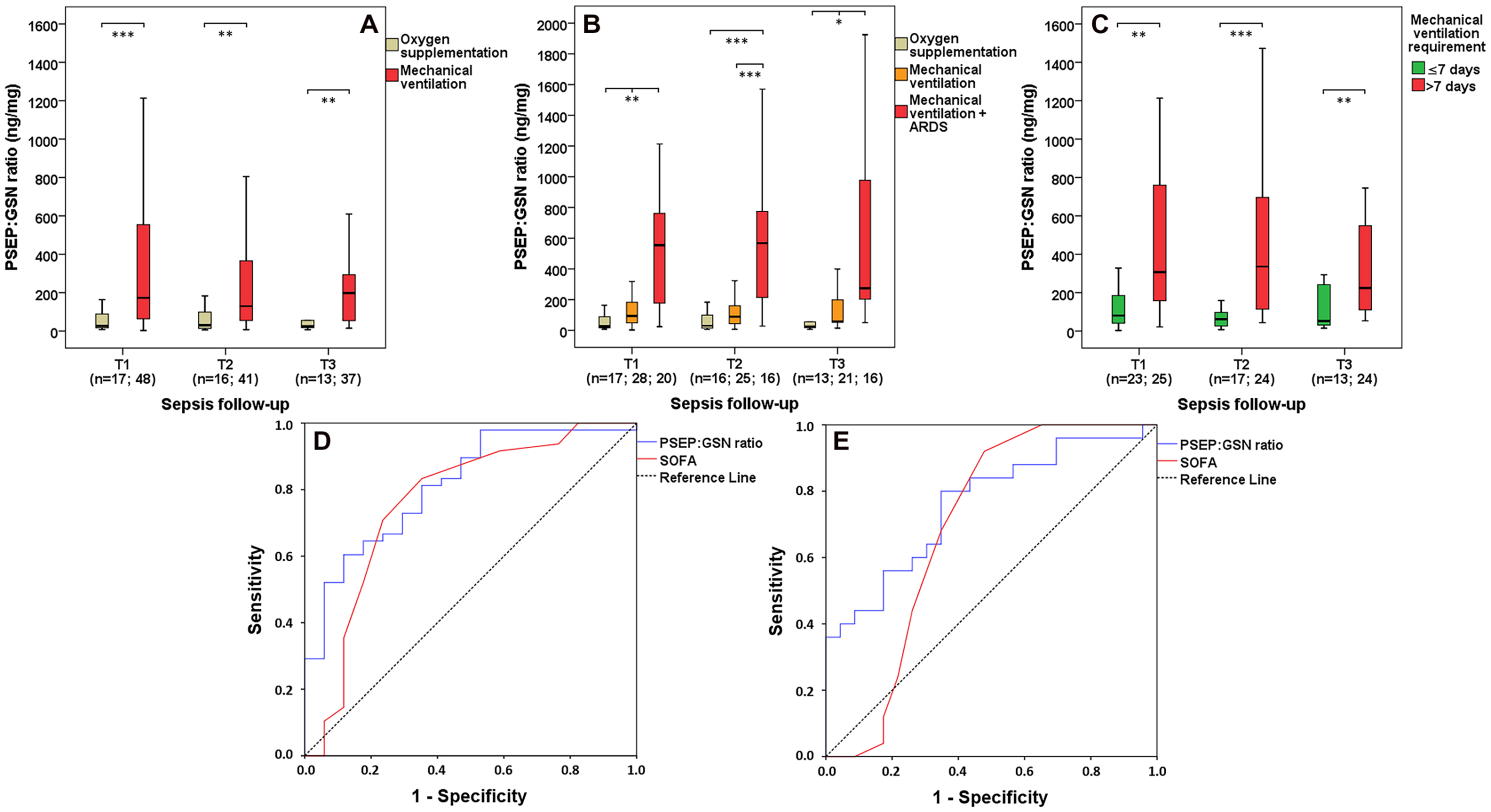
PSEP:GSN ratio in septic patients based on requirements of respiratory support. PSEP:GSN ratios of septic patients with requirements of oxygen supplementation and mechanical ventilation **(A)**, with the latter group having ARDS **(B)** during follow-up. PSEP:GSN ratios of septic patients having shorter (≤7 days) and longer (>7 days) requirement of mechanical ventilation during follow-up **(C)**. Receiver operating characteristic (ROC) curves of admission parameters for distinguishing septic patients needing oxygen supplementation from mechanical ventilation **(D)** along with discerning septic patients’ shorter (≤7 days) and longer (>7 days) requirement of mechanical ventilation **(E)**. Time points: T1: within 12 h after admission; T2: second day; T3: third day. ARDS, acute respiratory distress syndrome; PSEP, presepsin; PSEP:GSN, presepsin:gelsolin ratio; PCT, procalcitonin; SOFA, Sequential Organ Failure Assessment score. n: sample number. *p < 0.05; **p < 0.01; ***p < 0.001.

Septic patients with demand for mechanical ventilation showed significantly greater PSEP:GSN ratios than septic patients with oxygen supplementation requirement at T1 (median: 26.9 vs. 173.2 ng/mg, *p* < 0.001), T2 (median: 30.5 vs. 129.5 ng/mg, *p* = 0.002) and T3 (median: 25.4 vs. 198.5 ng/mg, *p* = 0.001; Figure 4A). In contrast to septic patients supported with oxygen supplementation, this elevating trend of PSEP:GSN ratio was even more explicit among septic patients treated with mechanical ventilation, if they developed moderate or severe stage ARDS during follow-up (T1 median: 26.9 vs. 94.2 vs. 554.8 ng/mg, *p* = 0.007; T2 median: 30.5 vs. 89.1 vs. 567.3 ng/mg, *p* < 0.001; T3 median: 25.4 vs. 58.6 vs. 273.6 ng/mg, *p* = 0.029; Figure 4B). Furthermore, septic patients needing mechanical ventilation longer than 7 consecutive days had significantly higher PSEP:GSN ratios than septic patients with shorter (≤7 days) demand for mechanical ventilation at T1 (median: 80.6 vs. 307.1 ng/mg, *p* = 0.002), T2 (median: 62.1 vs. 336.1 ng/mg, *p* < 0.001) and T3 (median: 52.2 vs. 224.3 ng/mg, *p* = 0.004) as well (Figure 4C). For differentiating septic patients with oxygen supplementation from patients with mechanical ventilation requirement, first-day AUC values were the following: PSEP:GSN ratio: 0.814 (*p* < 0.001); SOFA: 0.763 (*p* = 0.001). Derived cut-off values were: PSEP:GSN ratio: 68.8 ng/mg (sensitivity: 72.9%; specificity: 70.6%); SOFA: 9.5 (sensitivity: 70.8%; specificity: 76.5%; Figure 4D). For distinguishing septic patients with shorter (≤7 days) from longer (>7 days) demand for mechanical ventilation, first-day AUC values were as follows: PSEP:GSN ratio: 0.762 (*p* = 0.002); SOFA: 0.692 (*p* = 0.023). Derived cut-off values were: PSEP:GSN ratio: 134.3 ng/mg (sensitivity: 80.0%; specificity: 65.2%); SOFA: 10.5 (sensitivity: 68.0%; specificity: 65.2%; Figure 4E). Additional data regarding the ROC curve analysis of septic patients are presented in Supplementary Table 2.

### Monitoring presepsin:gelsolin ratio in septic non-AKI and sepsis-related AKI patients

**Figure 5:**
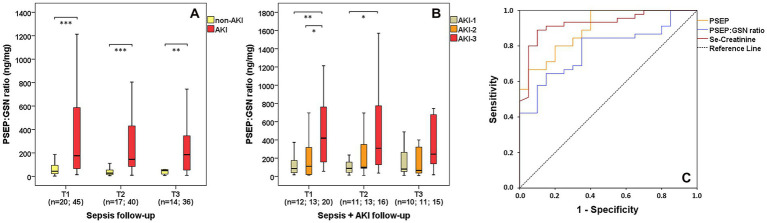
PSEP:GSN ratio in sepsis-related AKI. PSEP:GSN ratios of septic non-AKI and sepsis-related AKI patients **(A)** during follow-up. PSEP:GSN ratios of the individual sepsis-related AKI stages **(B)** during follow-up. Receiver operating characteristic (ROC) curves of admission laboratory parameters for distinguishing septic non-AKI from sepsis-related AKI state **(C)**. AKI, acute kidney injury; PSEP, presepsin; PSEP:GSN, presepsin:gelsolin ratio; PCT, procalcitonin. Time points: T1: within 12 h after admission; T2: second day; T3: third day. n: sample number. *p < 0.05; **p < 0.01; ***p < 0.001.

Sepsis-related AKI patients had substantially higher PSEP:GSN ratios than septic non-AKI patients at T1 (median: 43.6 vs. 176.1 ng/mg, *p* < 0.001), T2 (median: 27.5 vs. 145.1 ng/mg, *p* < 0.001) and T3 (median: 49.5 vs. 185.4 ng/mg, *p* = 0.009) as well (Figure 5A). Furthermore, PSEP:GSN ratios were even more increased between patients in AKI-1 and AKI-3 stage at T1 (median: 85.8 vs. 419.5 ng/mg, *p* = 0.006) and T2 (median: 87.6 vs. 308.8 ng/mg, *p* = 0.011), while a difference was also observed between patients in AKI-2 and AKI-3 stage at T1 (median: 111.1 vs. 419.5 ng/mg, *p* = 0.043; Figure 5B). For discerning all sepsis-related AKI patients from septic non-AKI patients, AUC value of first-day PSEP:GSN ratio (*p* < 0.001) was slightly lower than PSEP (*p* < 0.001) and se-creatinine (*p* < 0.001; Figure 5C; Table 2).

### Correlations

Quantitative data from all sample collection time points were used for calculating correlations. PSEP:GSN ratio showed strong correlation (*p* < 0.001) with PSEP (*ρ* = 0.924). Moderate correlations (*p* < 0.001) were found between PSEP:GSN ratio and se-urea (*ρ* = 0.720), se-creatinine (*ρ* = 0.611), hs-CRP (*ρ* = 0.573), PCT (*ρ* = 0.576) and WBC (*ρ* = 0.452), along with APACHE II (*ρ* = 0.759), SAPS II (*ρ* = 0.743) and SOFA (*ρ* = 0.741) clinical scores. PSEP:GSN ratio showed negative correlations (*p* < 0.001) with se-TP (*ρ* = −0.439), se-albumin (*ρ* = −0.667), and GSN (*ρ* = −0.853). In addition, PSEP had a moderate correlation (*p* < 0.001) to se-screatinine (*ρ* = 0.694). No further associations were observed with other inflammatory or clinical parameters.

## Discussion

One of the main focuses of our study was to describe the time course of PSEP:GSN ratio among non-septic and septic patients. In contrast to controls, significantly elevated PSEP:GSN ratios were detected in non-septic and septic patients. First-day PSEP:GSN ratios showed good performance compared with SOFA score and PSEP levels regarding the diagnosis of sepsis.

Moderate correlations were observed between PSEP:GSN ratio and the conventional inflammatory markers (hs-CRP, PCT). Regarding 10-day mortality data, PSEP:GSN ratios were substantially lower in survivors than non-survivors during follow-up, while the prognostic performance of PSEP:GSN ratio was similar to the widely used clinical scores (APACHE II, SAPS II, SOFA). These results suggest that PSEP:GSN ratio could also be a useful marker regarding the short-term mortality prediction of sepsis, yet the prognostic performance of PSEP levels was markedly inferior as opposed to the conventional clinical prognostic scores. Our results are slightly inconsistent with evidence from other multi-center studies showing better prognostic performance of PSEP in sepsis (14–16, 19, 40).

Regarding sepsis-related hemodynamic instability, increasing PSEP:GSN ratios were in good agreement with the dosage and the duration of vasopressor requirement in septic patients. Moreover, PSEP:GSN ratios were also higher in patients with septic shock than in septic patients without shock. First-day PSEP:GSN ratios also showed acceptable performance in relation to the SOFA score regarding the diagnosis of septic shock and the length of vasopressor requirement in sepsis. Concerning sepsis-related respiratory insufficiency, significantly elevated PSEP:GSN ratios were observed in patients needing mechanical ventilation compared with patients receiving oxygen supplementation. This increase was even more explicit in mechanically ventilated patients with (at least) moderate ARDS, while higher PSEP:GSN ratios were also associated with prolonged mechanical ventilation requirement in septic patients. First-day PSEP:GSN ratios performed relatively well in contrast to the SOFA score regarding the requirement and duration of mechanical ventilation in sepsis. Our results suggest that PSEP:GSN ratio could be a useful complementary marker besides the routinely used SOFA score, as the elevation of this parameter seems to have a good correlation with the progress of inflammation while also providing information about the patient’s actin scavenger capacity. Furthermore, the substantial increment of PSEP:GSN ratio may also indicate the need for prolonged organ support treatment in sepsis (6, 7).

As we previously observed elevated urinary actin levels in sepsis-related AKI, we also found that PSEP:GSN ratios were higher in sepsis-related AKI patients compared with septic non-AKI patients, especially in AKI-3 stage septic patients needing RRT (33). This tendency was the same when investigating PSEP levels among control, non-septic and septic patients. In accordance with previous studies, our results show a similarly increasing tendency of PSEP levels in sepsis and in sepsis-related AKI (14–21).

However, first-day se-creatinine had better performance than PSEP and PSEP:GSN ratio in the diagnosis of sepsis-related AKI. As se-creatinine only reflects the decreased glomerular filtration rate, our results suggest that PSEP:GSN ratio provides a more complex information regarding the patient’s condition and the overall organ dysfunction during sepsis. Therefore a growing body of evidence indicates that GSN (or its fragments) could also appear in the urine. Some studies found elevated urinary GSN levels (using western blot) in animal models of cisplatin/gentamicin-induced AKI, while urinary GSN was also investigated in patients with focal segmental glomerulosclerosis and rheumatoid arthritis as well (41–43). As far as we are aware, this is the first study to examine PSEP:GSN ratio in sepsis, therefore we did not have any other study for reference in this field. Additional investigation with extended case numbers may clarify the usefulness of PSEP:GSN ratio in the diagnosis of sepsis-related AKI.

Since GSN has a protective role by being an actin scavenger protein, numerous studies reported decreasing serum GSN concentrations in various clinical conditions (e.g., trauma, acute liver failure, myocardial infarction, sepsis) (27, 29–31). Our previous studies also showed declining serum GSN levels in sepsis and septic shock which were associated with increasing mortality rates (32, 37, 38). As a result, we also found significantly elevated PSEP:GSN ratios in septic patients, especially in severe sepsis-related organ dysfunctions (hemodynamic instability, respiratory insufficiency, AKI).

Our study has some limitations. To the best of our knowledge, PSEP:GSN ratio had not been explored before in sepsis, thus we aimed to be the first to examine this interesting area of clinical research. Therefore, no sample size or statistical power calculations were carried out prior to the study. We had limited capacities for consecutive patient enrollment, since our study was carried out as a single center study (16 bedded central ICU). Septic patients with severe organ dysfunction were more frequently admitted to our ICU being a regional center for critical care. Non-parametric tests (e.g., Mann–Whitney U test) may reduce the power of comparison, however, they could be applied adequately despite working with unequal sample sizes among control, non-septic and septic patient groups. The majority of patients were admitted at night or in the late afternoon before the actual first-day sample collection resulting in a slightly variable time interval (within 12 h) before taking the first sample. It is difficult to establish the timing of organ dysfunction in septic patients, therefore, the onset of sepsis-related organ dysfunctions was determined within 24 h after ICU admission. We are aware of the concern that outpatients are a difficult control group for ICU patients, yet we aimed to establish a reference range for PSEP:GSN ratios in patients without inflammation.

In the future, we should extend the number of critically ill patients due to the heterogeneity of sepsis while also prolonging the sample collection period to 5–10 days as well. Since there are no commercially available rapid diagnostic kits for GSN measurements, the development of an efficient point of care test would facilitate the prompt determination of PSEP:GSN ratio in routine clinical practice.

In conclusion, the present study demonstrated the diagnostic and prognostic utility of PSEP:GSN levels among non-septic and septic patients while also investigating the latter group based on the occurrence of sepsis-related organ dysfunctions including hemodynamic instability, respiratory insufficiency and AKI. Its diagnostic performance was acceptable in differentiating sepsis vs. septic shock and oxygen supplementation vs. mechanical ventilation requirement compared with the routinely used SOFA score. Furthermore, its prognostic ability was also promising regarding the length of vasopressor and mechanical ventilation requirement in sepsis which could help clinicians in the assessment of the patients’ condition. PSEP:GSN ratio could yield valuable information regarding the extent of inflammation and the simultaneous depletion of the patient’s scavenger capacity during sepsis. Further investigations with extended sampling periods and larger study populations are warranted to clarify the importance of PSEP:GSN ratio in sepsis.

## Data availability statement

The original contributions presented in the study are included in the article/Supplementary material, further inquiries can be directed to the corresponding author/s.

## Ethics statement

The studies involving human participants were reviewed and approved by the Regional Research Ethics Committee of the University of Pécs (no. 4327.316-2900/KK15/2011) conforming to the 7th revision of the Helsinki Declarations (2013). The patients/participants provided their written informed consent to participate in this study.

## Author contributions

DR took responsibility for sample and data analysis along with drafting the manuscript. PK was responsible for conceptualization and study design. ZH-S and BS participated in sample and data collection while providing assistance during blood sample analysis. AM, GW, TK, and DM were responsible for funding acquisition, approving data analysis and revision of the manuscript. All authors contributed to the article and approved the submitted version.

## Funding

The present research was funded by the 2020-4.1.1.-TKP2020 project (Thematic Excellence Program 2020 - National Excellence Subprogram of the Hungarian Ministry for Innovation and Technology) and partially by the European Union, grant number: RRF-2.3.1-21-2022-00012 “National Laboratory on Human Reproduction.” This work was also supported by the EFOP 3.6.1-16-2016-00004 project (Comprehensive Development for Implementing Smart Specialization Strategies) of the University of Pécs, Hungary. ZH-S was supported by the University of Pécs, Medical School, Hungary grant (KA-2019-36).

## Conflict of interest

The authors declare that the research was conducted in the absence of any commercial or financial relationships that could be construed as a potential conflict of interest.

## Publisher’s note

All claims expressed in this article are solely those of the authors and do not necessarily represent those of their affiliated organizations, or those of the publisher, the editors and the reviewers. Any product that may be evaluated in this article, or claim that may be made by its manufacturer, is not guaranteed or endorsed by the publisher.

## Abbreviations

ICU: intensive care unit
APACHE II: Acute Physiology and Chronic Health Evaluation II score
SAPS II: Simplified Acute Physiology Score II
SOFA: Sequential Organ Failure Assessment score
SSC: Surviving Sepsis Campaign
MODS: multiple organ dysfunction syndrome
PiCCO: Pulse Index Continuous Cardiac Output
ARDS: acute respiratory distress syndrome
AKI: acute kidney injury
RRT: renal replacement therapy
KDIGO: Kidney Disease Improving Global Outcomes
hs-CRP: high-sensitivity C-reactive protein
PCT: procalcitonin
PSEP: presepsin
GSN: gelsolin
PSEP:GSN: presepsin:gelsolin ratio
