# Supplementary material for: Presepsin: gelsolin ratio, as a promising marker of sepsis-related organ dysfunction: a prospective observational study
Source: Front Med (Lausanne). 2023 May 5;10:1126982. doi: 10.3389/fmed.2023.1126982 (PMC10196472; doi:10.3389/fmed.2023.1126982)
Supplement: Supplementary file 1 [file Table_1.docx]

**Supplementary Table 1.**

**Clinical and laboratory data of septic patients.**

|  | **Sepsis without MODS (n=24)** | **Sepsis + MODS (n=41)** | **p value** |
| --- | --- | --- | --- |
| Age (years) | 69 (59 - 79) | 66 (56 - 72) | 0.286 |
| Males, n (%) | 15 (62.5) | 28 (68.3) | 0.634 |
| **Major admission laboratory parameters** | | | |
| se-albumin (g/L) | 24.8 (21.5 - 29.1) | 22.1 (18.4 - 25.4) | 0.035 |
| se-creatinine (µmol/L) | 105 (84 - 155) | 196 (149 - 340) | <0.001 |
| se-bilirubin (µmol/L) | 5.6 (4.5 - 9.1) | 18.8 (7.2 - 38.9) | <0.001 |
| PLT (G/L) | 235 (156 - 298) | 182 (94 - 312) | 0.219 |
| hs-CRP (mg/L) | 273.6 (156.9 - 342.5) | 290.2 (194.6 - 389.6) | 0.505 |
| PCT (ng/mL) | 7.3 (2.4 - 28.6) | 22.3 (6.9 - 59.8) | 0.046 |
| PSEP (pg/mL) | 470.5 (354 - 687) | 1878 (1071 - 4136) | <0.001 |
| GSN (mg/L) | 17.3 (8.4 - 30.9) | 10.3 (5.5 - 17.4) | 0.017 |
| PSEP:GSN ratio (ng/mg) | 26.9 (18.2 - 77.1) | 182.7 (95.9 - 609.4) | <0.001 |
| pl-lactate (mmol/L) | 1.5 (0.8 - 2.1) | 2.4 (1.8 - 4.1) | 0.002 |
| **Cause of admission** | | | |
| Internal medicine origin, n (%) | 9 (37.5) | 8 (19.5) | 0.147 |
| Surgical origin, n (%) | 15 (62.5) | 33 (80.5) | 0.111 |
| **Clinical prognostic parameters** | | | |
| APACHE II score | 14 (11 - 19) | 22 (19 - 26) | <0.001 |
| SAPS II score | 37 (33 - 43) | 51 (45 - 57) | <0.001 |
| SOFA score | 8 (6 - 9) | 11 (10 - 13) | <0.001 |
| ICU treatment days | 4 (3 - 9) | 10 (6 - 15) | 0.004 |
| 10-day mortality, death (%) | 1 (4.2) | 17 (41.5) | 0.001 |
| AKI requiring RRT, n (%) | 2 (8.3) | 13 (31.7) | 0.036 |

Continuous variables are shown as median (25th - 75th percentiles) and categorical variables are expressed as a number (percentage). Mann-Whitney U and Chi-square tests were performed for data comparison between patient groups. Level of significance was set at p<0.05. Abbreviations: ICU: intensive care unit; MODS: multiple organ dysfunction syndrome; PLT: platelet count; PCT: procalcitonin; hs-CRP: high-sensitivity C-reactive protein; PSEP: presepsin; GSN: gelsolin; PSEP:GSN: presepsin:gelsolin ratio; AKI: acute kidney injury; RRT: renal replacement therapy; APACHE II: Acute Physiology and Chronic Health Evaluation II score; SAPS II: Simplified Acute Physiology Score II; SOFA: Sequential Organ Failure Assessment score.

**Additional clinical and microbiological data of septic patients.**

|  | **Sepsis without MODS (n=24)** | **Sepsis + MODS (n=41)** | **p value** |
| --- | --- | --- | --- |
| **Major therapeutic requirements, n (%)** | | | |
| Vasopressor support | 14 (58.3) | 40 (97.6) | <0.001 |
| Oxygen supplementation | 13 (54.2) | 4 (9.8) | <0.001 |
| Mechanical ventilation | 11 (45.8) | 37 (90.2) | <0.001 |
| Hydrocortisone supplementation | 18 (75.0) | 35 (85.4) | 0.299 |
| **Focus of infection, n (%)** | | | |
| Respiratory | 5 (20.8) | 11 (26.8) | 0.588 |
| Urogenital | 4 (16.7) | 5 (12.2) | 0.715 |
| Abdominal | 7 (29.1) | 12 (29.3) | 0.993 |
| Soft tissue/Bones | 4 (16.7) | 5 (12.2) | 0.715 |
| Mixed | 4 (16.7) | 8 (19.5) | 0.775 |
| **Identified pathogens, n (%)** | | | |
| Unidentified | 13 (54.2) | 7 (17.1) | 0.021 |
| Gram-positive bacteria | 2 (8.3) | 6 (14.6) | 0.614 |
| Gram-negative bacteria | 1 (4.2) | 6 (14.6) | 0.331 |
| Fungi | 2 (8.3) | 2 (4.9) | 0.619 |
| Mixed | 6 (25.0) | 20 (48.8) | 0.024 |

Categorical variables are expressed as a number (percentage). Chi-square tests were performed for data comparison between patient groups. Level of significance was set at p<0.05. Abbreviations: MODS: multiple organ dysfunction syndrome.
