# Supplementary material for: Presepsin: gelsolin ratio, as a promising marker of sepsis-related organ dysfunction: a prospective observational study
Source: Front Med (Lausanne). 2023 May 5;10:1126982. doi: 10.3389/fmed.2023.1126982 (PMC10196472; doi:10.3389/fmed.2023.1126982)
Supplement: Supplementary file 2 [file Table_2.docx]

**Supplementary Table 2.**

**Receiver operating characteristic (ROC) curve analysis of septic patients.**

| **Variable** | **AUC (95% CI)** | **Standard error*** | **Sens.**  **(%)** | **Spec.**  **(%)** | **Cut-off value** | **p value** |
| --- | --- | --- | --- | --- | --- | --- |
| **Sepsis (n=38) vs. Septic shock (n=27)** | | | | | | |
| PSEP:GSN ratio (ng/mg) | 0.824 (0.720 - 0.927) | 0.053 | 70.4 | 78.9 | 161.2 | <0.001 |
| SOFA score | 0.818 (0.715 - 0.920) | 0.052 | 70.4 | 76.3 | 10.5 | <0.001 |
| **Comparison of ROC curves (DeLong test significance levels):** PSEP:GSN vs. SOFA (p=0.932) | | | | | | |
| **Shorter (≤5 days) (n=25) vs. longer (>5 days) (n=29) vasopressor support in sepsis** | | | | | | |
| PSEP:GSN ratio (ng/mg) | 0.821 (0.705 - 0.937) | 0.059 | 93.1 | 68.0 | 91.7 | <0.001 |
| SOFA score | 0.698 (0.546 - 0.850) | 0.077 | 89.7 | 56.0 | 9.5 | 0.013 |
| **Comparison of ROC curves (DeLong test significance levels):** PSEP:GSN vs. SOFA (p=0.155) | | | | | | |
| **Oxygen supplementation (n=17) vs. mechanical ventilation (n=48) requirement in sepsis** | | | | | | |
| PSEP:GSN ratio (ng/mg) | 0.814 (0.699 - 0.929) | 0.059 | 72.9 | 70.6 | 68.8 | <0.001 |
| SOFA score | 0.763 (0.612 - 0.914) | 0.077 | 70.8 | 76.5 | 9.5 | 0.001 |
| **Comparison of ROC curves (DeLong test significance levels):** PSEP:GSN vs. SOFA (p=0.608) | | | | | | |
| **Shorter (≤7 days) (n=23) vs. longer (>7 days) (n=25) mechanical ventilation requirement in sepsis** | | | | | | |
| PSEP:GSN ratio (ng/mg) | 0.762 (0.627 - 0.896) | 0.069 | 80.0 | 65.2 | 134.3 | 0.002 |
| SOFA score | 0.692 (0.527 - 0.857) | 0.084 | 68.0 | 65.2 | 10.5 | 0.023 |
| **Comparison of ROC curves (DeLong test significance levels):** PSEP:GSN vs. SOFA (p=0.439) | | | | | | |

Receiver operating characteristic (ROC) curve analysis of admission laboratory and clinical parameters for distinguishing sepsis from septic shock along with differentiating shorter (≤5 days) from longer (>5 days) requirement of vasopressor support, while also discerning septic patients’ requirement of oxygen supplementation from mechanical ventilation and their shorter (≤7 days) or longer (>7 days) requirement of mechanical ventilation. Abbreviations: AUC: area under the curve; 95% CI: 95% Confidence interval; Sens.: Sensitivity; Spec.: Specificity; PSEP: presepsin; PSEP:GSN: presepsin:gelsolin ratio; PCT: procalcitonin; SOFA: Sequential Organ Failure Assessment score. *DeLong et al., 1988.
